# Diminazene Aceturate Improves Cardiac Fibrosis and Diastolic Dysfunction in Rats with Kidney Disease

**DOI:** 10.1371/journal.pone.0161760

**Published:** 2016-08-29

**Authors:** Elena Velkoska, Sheila K. Patel, Karen Griggs, Louise M. Burrell

**Affiliations:** Department of Medicine, The University of Melbourne, Austin Health, Heidelberg, Victoria, Australia; University Medical Center Utrecht, NETHERLANDS

## Abstract

Angiotensin converting enzyme (ACE) 2 is a negative regulator of the renin angiotensin system (RAS) through its role to degrade angiotensin II. In rats with subtotal nephrectomy (STNx), adverse cardiac remodelling occurs despite elevated cardiac ACE2 activity. We hypothesised that diminazene aceturate (DIZE), which has been described as having an off-target effect to activate ACE2, would have beneficial cardiac effects in STNx rats. STNx led to hypertension, diastolic dysfunction, left ventricular hypertrophy, cardiac fibrosis, and increased cardiac ACE, ACE2, Ang II and Ang 1–7 levels. Cardiac gene expression of ADAM17 was also increased. In STNx, two-weeks of subcutaneous DIZE (15mg/kg/d) had no effect on blood pressure but improved diastolic dysfunction and cardiac fibrosis, reduced ADAM17 mRNA and shifted the cardiac RAS balance to a cardioprotective profile with reduced ACE and Ang II. There was no change in cardiac ACE2 activity or in cardiac Ang 1–7 levels with DIZE. In conclusion, our results suggest that DIZE exerts a protective effect on the heart under the pathological condition of kidney injury. This effect was not due to improved kidney function, a fall in blood pressure or a reduction in LVH but was associated with a reduction in cardiac ACE and cardiac Ang II levels. As *in vitro* studies showed no direct effect of DIZE on ACE2 or ACE activity, the precise mechanism of action of DIZE remains to be determined.

## Introduction

The prevalence of kidney disease is increasing worldwide and cardiovascular disease is the major cause of morbidity and mortality [1, 2]. Activation of the renin angiotensin system (RAS) plays an important role in the development and progression of kidney and heart disease. In the classic RAS, angiotensin converting enzyme (ACE) converts angiotensin (Ang) I to Ang II, which mediates its adverse effects via the angiotensin type 1 receptor [3]. RAS blockade is recommended as first-line therapy in patients with kidney disease to improve outcomes [4], but as kidney and cardiac disease continue to progress, new therapeutic approaches are needed.

In the alternate arm of the RAS [5, 6], ACE2 counterbalances an activated ACE/Ang II pathway through degradation of Ang II, and generation of the antifibrotic peptide, Ang 1–7 [7, 8]. Increasing evidence suggests that the relative balance between the deleterious ACE/Ang II pathway and the protective ACE2/Ang 1–7 pathway is an important determinant of tissue injury. In kidney disease secondary to subtotal nephrectomy (STNx), kidney ACE and Ang II are increased [9–13] and kidney ACE2 activity is decreased [12, 14]. These findings have led to strategies to replenish ACE2 using recombinant ACE2 or to activate ACE2 using compounds such as diminazene aceturate (DIZE) [15]. Both approaches have beneficial effects on the kidney in experimental kidney disease. Recombinant human ACE2 prevented Ang II induced kidney disease and tubulointerstitial fibrosis [16] and slowed the progression of diabetic nephropathy through a reduction in renal Ang II and increased Ang 1–7 levels [17]. DIZE had reno-protective effects after renal ischemia/reperfusion injury through enhancement of antioxidant activity [18], and we reported that short-term DIZE decreased kidney cortical ACE activity and ameliorated the reduction in kidney ACE2 expression and activity in STNx rats [13].

Kidney disease is also associated with activation of the cardiac RAS and cardiac damage. We previously reported that STNx rats have increased cardiac ACE activity and adverse cardiac remodelling despite elevations in cardiac ACE2 activity [19, 20]. We also showed that ramipril lowered blood pressure and inhibited cardiac ACE activity, and that these changes were associated with a reduction in LVH and cardiac ACE2 activity [19]. In the current paper, we hypothesised that DIZE which has been described as having an off-target effect to activate ACE2, would have beneficial cardiac effects in STNx rats. Full cardiac hemodynamic data was available in a subset of rats from our previously published study [13] along with cardiac tissue. Therefore, the focus of this paper was to investigate the effect of short-term DIZE on cardiac structure and function, cardiac ACE/ACE2 gene expression/activity and cardiac angiotensin peptide (Ang II/Ang 1–7) levels in STNx rats with an activated RAS and in Control rats with a balanced RAS. In addition, we assessed gene expression of cardiac ADAM17 [21], a proteinase that is responsible for the cleavage or “shedding” of the catalytically active ectodomain of ACE2 from the cell membrane.

## Materials and Methods

### Experimental Protocol

Experimental procedures were performed in accordance with the National Health and Medical Research Council of Australia guidelines for animal experimentation and were approved by the Animal Ethics Committee, Austin Health. Female Sprague Dawley (SD) rats (body weight of 190-210g) were housed in a 12:12h light-dark cycle, with *ad libitum* food containing 0.4–0.6% NaCl (Norco) and water. STNx (*n* = 16) was performed as described previously [12, 13, 19, 20], with a right nephrectomy, and ligation of all but one of the extra-renal branches of the left renal artery. STNx rats were randomly allocated to the presumed ACE2 activator DIZE (2 weeks s.c. 15mg/kg/day, *n* = 8) via osmotic minipump (Model # 2002, Alzet, Cupertino, CA, USA) or Vehicle (*n* = 8), which was implanted at the time of STNx surgery. We used the same dose and mode of delivery of DIZE as in previously published studies [13, 22, 23]. Control rats received DIZE (2 weeks s.c. 15mg/kg/day, *n* = 8) or Vehicle (*n* = 8).

On day 14, rats were anaesthetised with intraperitoneal (i.p.) sodium pentobarbitone (60 mg/kg/body weight), and cardiac haemodynamics were determined using a micro-tipped pressure transducer catheter (Millar, 1.5F) inserted into the left carotid artery and advanced into the left ventricle (LV) as described previously [14, 20]. Data were stored and analysed using Millar conductance data acquisition and analysis software, and heart rate, systolic blood pressure and maximal and minimal rate of ventricular contraction (±dP/dt) were determined [14, 20]. We also measured the time constant of isovolumic relaxation (Tau), which measures active relaxation, with higher values of Tau implying impaired relaxation [24].

Rats were killed by a lethal dose of sodium pentobarbitone, decapitated and trunk blood collected into either lithium heparin tubes or into EDTA tubes containing 20 μl/ml of blood of an enzyme inhibitor cocktail [50 mM EDTA, 0.2 M N-ethylmaleimide and 1–2 TIU (trypsin inhibitory units)/ml aprotinin made up in saline] [20]. After centrifugation, plasma was separated, snap-frozen and stored at −80°C. The heart was removed, weighed and the LV was transversely dissected into 3 pieces, with one piece fixed in 4% paraformaldehyde and embedded in paraffin for histopathology. The remainder of the LV was snap-frozen in isopentane and stored at -80°C for mRNA extraction, peptide analysis and activity assays.

### Drugs

Sodium pentobarbitone was obtained from Boehringer Ingelheim, Artarmon, NSW, Australia), DIZE from Sigma-Aldrich Australia.

### Biochemical analysis

Plasma creatinine (Cr) was measured using an autoanalyser (Beckman Instruments, Palo Alta, CA, USA).

### Determination of cardiac collagen

Cardiac (LV) paraffin sections 4μm thick were deparaffinized, rehydrated and stained with 0.1% Sirius Red (Polysciences Inc) in saturated picric acid (picrosirius red) for 1 hour, differentiated in 0.01% HCl for 30 seconds, and rapidly dehydrated. Interstitial collagen volume fraction was determined by measuring the area of stained tissue within a given field, excluding vessels, artefacts, minor scars or incomplete tissue fields; 15–20 fields were analysed per animal in a blinded manner [14, 20]. To measure perivascular collagen, all arteries in the LV section were analysed, and the whole artery including the adventitia was selected for assessment. For both interstitial and perivascular collagen, the area stained was calculated as a percentage of the total area within a given field [25, 26].

### Plasma and cardiac angiotensin peptides

Blood for the measurement of angiotensin peptides were collected and stored as described above. Frozen LV sections were homogenised in 5mM EDTA solution containing a protease inhibitor (P8340, Sigma Aldrich; components: 104 mM AEBSF [4-(2-Aminoethyl) benzenesulfonyl fluoride hydrochloride], 80μM aprotinin, 4mM bestatin, 1.4mM E-64, 2mM leupeptin and 1.5mM pepstatin A), frozen at -80°C to aid in cell disruption, thawed and centrifuged prior to assaying (Prosearch, Australia). Angiotensin peptide content is expressed per mg of protein content of each individual sample.

The radioimmunoassays for Ang II and Ang 1–7 have been described previously [12, 20]. The antibodies used for Ang II and Ang 1–7 were raised in rabbit and guinea pig respectively, and the specific radioisotopes, ^125^I-Ang II and ^125^I-Ang 1–7, were made by Prosearch (Melbourne, Australia). The intra- and inter-assay coefficients of variation were 7.6 and 8.3% for Ang II and 4.5 and 10% for Ang 1–7.

### *In vivo* cardiac ACE and ACE2 activity

LV membrane preparations were prepared as previously described [19], and ACE activity measured using an enzymatic assay [20]. Briefly, 100μg of membrane protein was incubated at 37°C with the ACE substrate hippuryl-His-Leu (1mM) in a total volume of 50μl in the presence and absence of EDTA (10μM) for 60 min. The rate of substrate cleavage was determined by comparison to a standard curve of the product His-Leu and expressed as nmole of substrate cleaved/mg of protein/hr.

LV ACE2 was measured using an enzymatic assay as previously described [19, 20]. Briefly, 100μg of membrane protein was incubated in duplicate with an ACE2 quenched fluorescent substrate (QFS), (7-methoxycoumarin-4-yl)-acetyl-Ala-Pro-Lys (2, 4-dintirophenyl); Auspep, Parkville, Victoria, Australia), as previously described [19, 20, 27]. The specific activity was determined using 100μM EDTA which has a similar effect to inhibit ACE2 as the specific ACE2 inhibitor, MLN4760 [28, 29]. In addition, as QFS can be cleaved by prolyl endopeptidase, an inhibitor of this enzyme, Z-Pro-prolinal (1μM) was included in all wells [30]. The rate of substrate cleavage was determined by comparison to a standard curve of the free fluorophore, 4-amino-methoxycoumarin (MCA; Sigma, MO, USA) and expressed as nmole of substrate cleaved/mg of protein/hr.

For plasma ACE and ACE2 activity, blood collected into heparinised tubes was centrifuged at 4°C and assayed as above. Results are expressed as nmole of substrate/ml of plasma/hr.

### *In vitro* effect of DIZE on cardiac (LV) ACE2 and ACE activity and ACE binding

Cardiac (LV) membranes from STNx (*n* = 4) and Control (*n* = 4) rats were incubated with increasing concentrations of DIZE (0.1mM, 0.1μM, 0.1nM) or vehicle. ACE and ACE2 activity was measured as described above and results expressed as nmole of substrate cleaved/mg of protein/hr.

The effect of DIZE on cardiac ACE binding was also assessed using *ex vivo* autoradiography on LV sections (20μm) using the specific ACE inhibitor radioligand ^125^I-MK351A (K_i_ = 30 pmol/l) [14, 19, 20]. Sections were incubated with serial dilutions of DIZE or the ACE inhibitor ramiprilat (10^−10^ to 10^−3^ mol/l) (*n* = 4 per concentration). Quantification of ACE binding density was performed using a microcomputer-imaging device (MCID, Imaging Research, UK) which measures the relative absorbance of the radioactive labelling.

### Cardiac (LV) ACE2 immunohistochemistry

Immunohistochemical staining for ACE2 (polyclonal antibody, T17, from Santa Cruz Biotechnology, Santa Cruz, CA, USA; diluted 1:100) was performed in rat LV sections as described previously [14, 19, 25]. Staining was quantified using computerized image analysis (Imaging Research, Linton, Cambridge, UK). All sections used for quantification were fixed, processed, sectioned and immunolabelled at the same time and under the same conditions to limit variability. Images were imported into the AIS Imaging program using a colour video camera and a standard light microscope (magnification ×20). The detection level threshold for positively stained areas (brown for 3,3-Diaminobenzidine staining) was set so that the processed image accurately reflected the positively stained areas as visualized by light microscopy on the unprocessed digital image. Myocyte ACE2 staining was determined by measuring the area of stained tissue within a given field, excluding vessels, artifacts, minor scars or incomplete tissue fields. Fifteen to 20 fields were analysed per animal. The percentage area of chromogen staining was determined by calculating the number of selected pixels (positively stained areas) as a percentage of the total area within a given field [26].

### Cardiac (LV) gene expression

Total RNA was isolated from the LV using the RNeasy fibrous kit method (Qiagen). cDNA was synthesized with a reverse transcriptase reaction using standard techniques (Superscript III kit; Life Technologies) as described previously [19, 20]. The primers and probes for ACE, ACE2 and brain natriuretic peptide (BNP) were designed using the software program Primer Express (PE Applied Biosystems). Collagen 1A (Col1A) and ADAM17 TaqMan gene expression assay was purchased from Applied Biosystems. Quantitative RT-PCR was performed using the TaqMan system based on real-time detection of accumulated fluorescence (7500 Real Time PCR System, Applied Biosystems, CA, USA). Gene expression was normalized to 18S VIC and reported as ratios compared with the level of expression in Control rats, which were given an arbitrary value of 1.

### Statistical Analysis

Data are presented as mean ± standard error of mean (SEM). *P* values were calculated using a two-way analysis of variance (ANOVA), followed by post hoc Bonferroni tests (GraphPad Prism 6) comparing effect of STNx (Control vs. STNx) and effect of treatment (Control vs Control+DIZE; STNx vs. STNx+DIZE). For data with unequal variance, results were log-transformed and analysed using Kruskal-Wallis test with Dunn’s multiple comparison test. The Pearson correlation coefficient was determined for the associations between variables using the data from untreated Control and STNx rats. Two-tailed *P*-values <0.05 were considered significant.

## Results

### Physiological, biochemical and plasma RAS parameters after STNx

Following STNx, rats had reduced body weight (*P<*0.01) and kidney impairment with elevated plasma Cr (*P<*0.001) compared to Controls (Table 1). Plasma ACE2 activity, Ang II and Ang 1–7 were increased in STNx rats (*P*<0.05), with no change in plasma ACE activity compared to Control rats. Treatment with DIZE did not alter circulating RAS components or improve kidney function in Control or STNx rats.

**Table 1:** Renal function and plasma RAS components.

	Control		Subtotal nephrectomy	
	Vehicle	DIZE	Vehicle	DIZE
		(15 mg/kg/day)		(15 mg/kg/day)
	(*n* = 8)	(*n* = 8)	(*n* = 8)	(*n* = 8)
Body weight (g)	239 ± 4	232 ± 3	214 ± 8**	210 ± 10
Plasma creatinine (μmol/L)	17 ± 1	16 ± 1	46 ± 5***	40 ± 2
**Plasma RAS Components**				
ACE activity (nmol/ml/hr)	1714 ± 67	1529 ± 116	1589 ± 98	1582 ± 143
ACE2 activity (nmol/ml/hr)	5.6 ± 0.6	5.3 ± 0.2	7.1 ± 0.4*	6.5 ± 0.4
Ang II (fmol/ml)	28.4 ± 3.4	29.6 ± 2.9	70.9 ± 18.4*	60.6 ± 11.2
Ang 1–7 (fmol/ml)	183 ± 16	196 ± 24	327 ± 50*	456 ± 72

Data expressed as mean ± SEM.

*P<0.05

**P<0.01

***P<0.001 disease effect (Control Vehicle vs. STNx Vehicle).

### Blood pressure and cardiac function and structure

**Fig 1:**
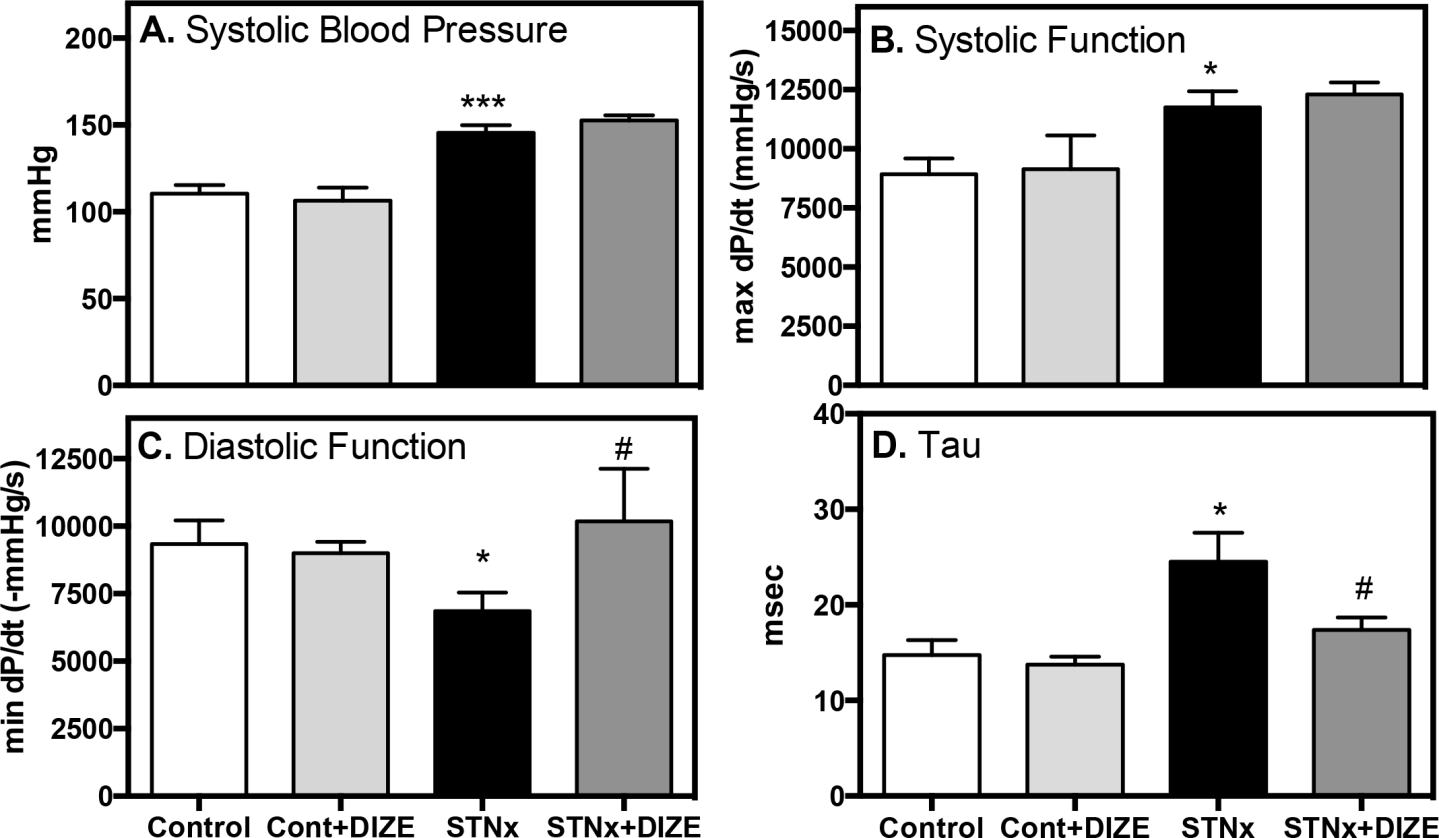
DIZE improved diastolic dysfunction in STNx. Systolic blood pressure (**A**), ventricular contractility (**B**), ventricular relaxation (**C**) and time constant of isovolumic relaxation (Tau; **D**) in Control (Cont) and subtotal nephrectomy (STNx) rats (*n* = 8/group). Data expressed as mean±SEM. *P<0.05, ***P<0.001 disease effect (Control vs. STNx) and # *P*<0.05 treatment effect (Vehicle vs. DIZE)

**Fig 2:**
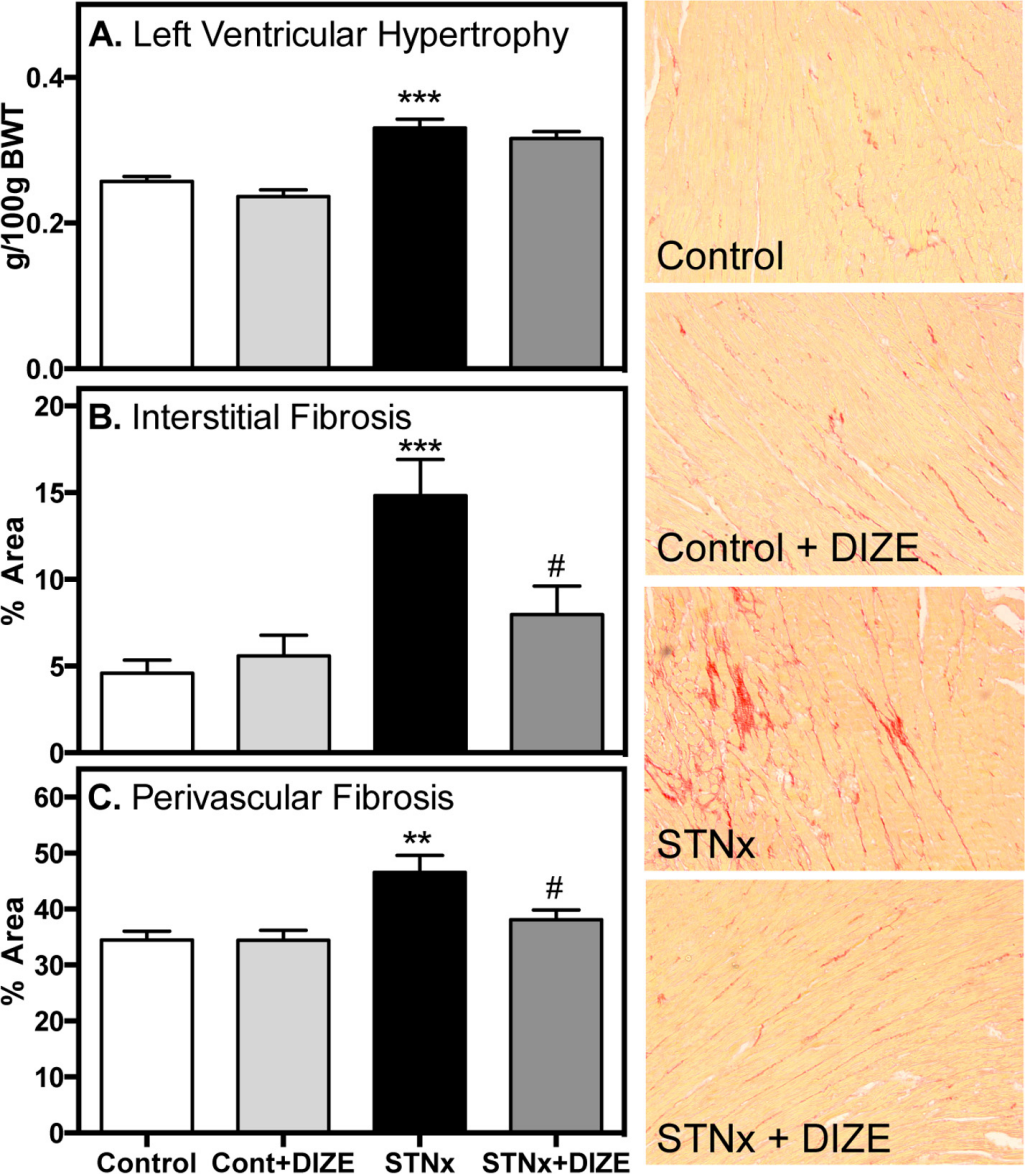
DIZE was associated with a reduction in cardiac fibrosis in STNx. Left ventricular hypertrophy (**A**), interstitial collagen (**B**) and perivascular collagen (**C**) in Control (Cont) and subtotal nephrectomy (STNx) rats (*n* = 8/group). Right hand panel consists of representative photomicrographs of left ventricular total collagen content (red staining) (magnification x200). Data expressed as mean±SEM. **P<0.01, ***P<0.001 disease effect (Control vs. STNx) and # *P*<0.05 treatment effect (Vehicle vs. DIZE)

STNx rats had increased systolic blood pressure (Fig 1A, *P*<0.001) and cardiac hypercontractility (Fig 1B, *P*<0.05). Diastolic dysfunction was present with impaired active relaxation shown by a reduction in min dP/dt (Fig 1C, *P*<0.05) and an increased time constant for isovolumic relaxation (Tau) (Fig 1D, *P*<0.05). In STNx, DIZE had no effect on blood pressure or contractility but improved diastolic function compared to vehicle treated rats (Fig 1C and 1D, *P*<0.05). STNx was associated with LV hypertrophy (LVH, Fig 2A, *P*<0.001) and significant interstitial (Fig 2B, *P*<0.001) and perivascular fibrosis (Fig 2C, *P*<0.01). DIZE reduced both interstitial and perivascular fibrosis (*P*<0.05), with no effect on LVH. DIZE had no effect on blood pressure, cardiac structure or function in Control rats.

### Cardiac (LV) gene expression

Cardiac ACE and ACE2 gene expression were increased in STNx rats compared to Control (both *P*<0.05), and were unchanged with DIZE (Table 2). Cardiac ADAM17 gene expression was also elevated in STNx rats and reduced with DIZE (Table 2). BNP mRNA expression, a marker of cardiac damage was increased in the LV of STNx rats (*P*<0.001) and reduced by DIZE (*P*<0.05). Col1A (*P*<0.00) gene expression was increased in the LV of STNx rats, and reduced by DIZE (*P*<0.05) in keeping with the reduction in collagen protein. In Control rats, DIZE had no effect on cardiac ACE, ACE2, BNP or Col1A gene expression.

**Table 2:** Cardiac (LV) gene expression.

	Control		Subtotal nephrectomy	
	Vehicle	DIZE	Vehicle	DIZE
		(15 mg/kg/day)		(15 mg/kg/day)
	(*n* = 8)	(*n* = 8)	(*n* = 8)	(*n* = 8)
ACE (arbitrary units)	1.00 ± 0.22	1.34 ± 0.24	1.99 ± 0.32*	1.69 ± 0.33
ACE2 (arbitrary units)	1.00 ± 0.13	1.16 ± 0.18	1.46 ± 0.17*	1.41 ± 0.12
BNP (arbitrary units)	1.00 ± 0.22	0.97 ± 0.19	4.13 ± 0.56***	2.56 ± 0.41^#^
Col1A (arbitrary units)	1.00 ± 0.23	1.48 ± 0.24	3.92 ± 0.54***	2.52 ± 0.37^#^
ADAM17 (arbitrary units)	1.00 ± 0.16	1.12 ± 0.14	1.80 ± 0.27*	1.13 ± 0.09^#^

Data expressed as mean±SEM.

*P<0.05

***P<0.001 disease effect (Control Vehicle vs. STNx Vehicle)

#*P*<0.05 treatment effect (Vehicle vs. DIZE)

### Cardiac (LV) RAS components

**Fig 3:**
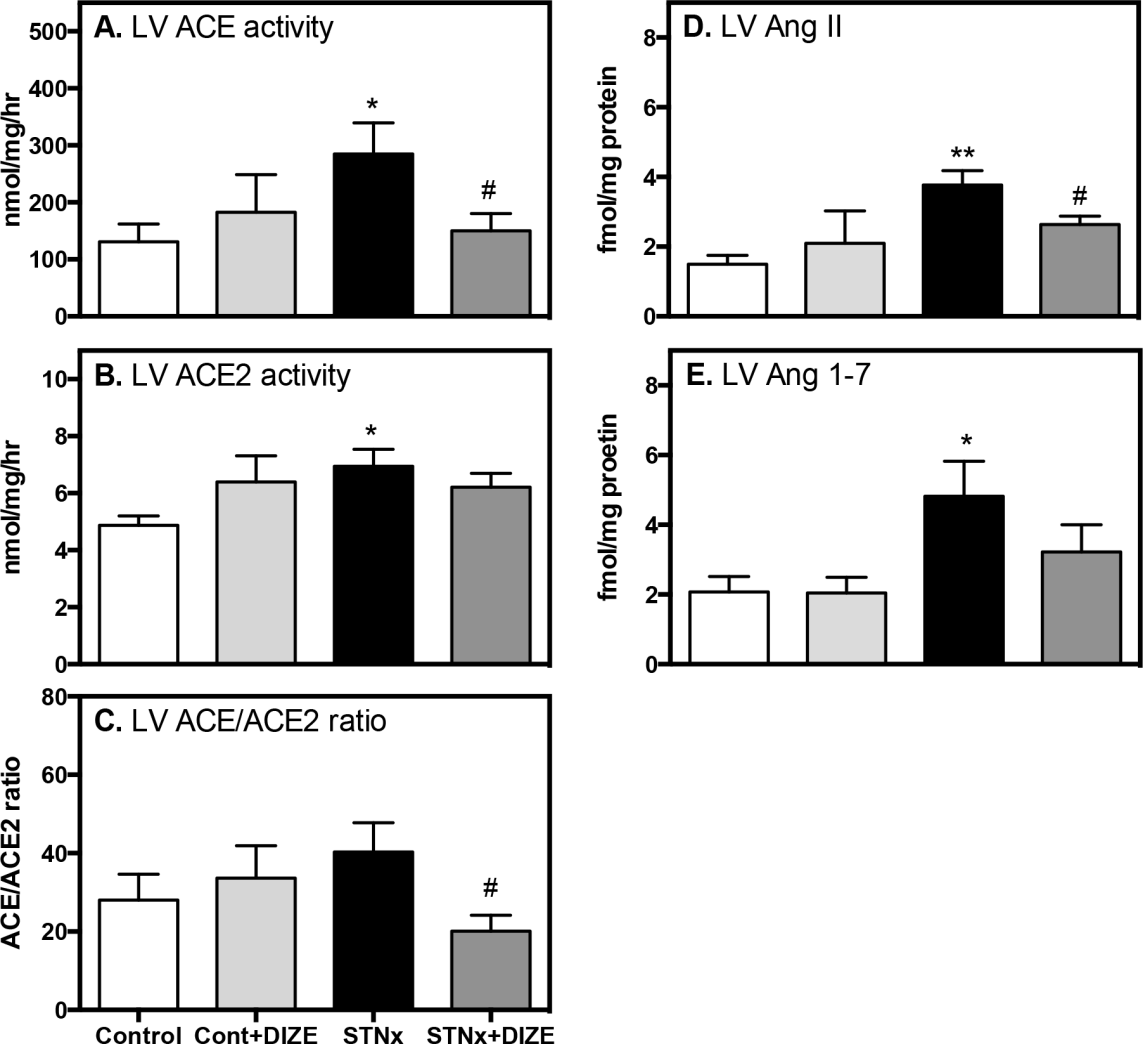
DIZE shifts the cardiac RAS balance to a cardioprotective profile in STNx. Left ventricular (LV) ACE (**A**) and ACE2 activity (**B**), ACE/ACE2 activity ratio (**C**), LV Ang II (**D**) and Ang 1–7 (**E**) peptide content in Control (Cont) and subtotal nephrectomy (STNx) rats (*n* = 8/group). Data expressed as mean ± SEM. *P<0.05 disease effect (Control vs. STNx) and # *P*<0.05 treatment effect (Vehicle vs. DIZE)

Cardiac ACE activity (Fig 3A) and ACE2 activity (Fig 3B) were increased in STNx rats compared to Control rats (both, *P*<0.05). Cardiac ACE2 protein was also elevated in STNx rat and unchanged with DIZE treatment (S1 Fig). In STNx, DIZE treatment was associated with a reduction in cardiac ACE activity (*P*<0.05), but no change in cardiac ACE2 activity compared to the untreated STNx group. This resulted in a decrease in the ACE/ACE2 activity ratio towards a more favourable and cardioprotective profile (Fig 3C, *P*<0.05). In Control rats, DIZE had no effect on cardiac ACE or ACE2 protein or activity.

Cardiac Ang II (Fig 3D; *P*<0.01) and Ang 1–7 (Fig 3E, *P*<0.05) were increased in STNx rats compared to Control. In STNx, DIZE treatment reduced cardiac Ang II levels (*P*<0.05) and Ang 1–7 levels remained unchanged. DIZE had no effect on cardiac Ang peptides in Control rats.

#### Circulating vs. cardiac tissue ACE and ACE2 activity

**Fig 4:**
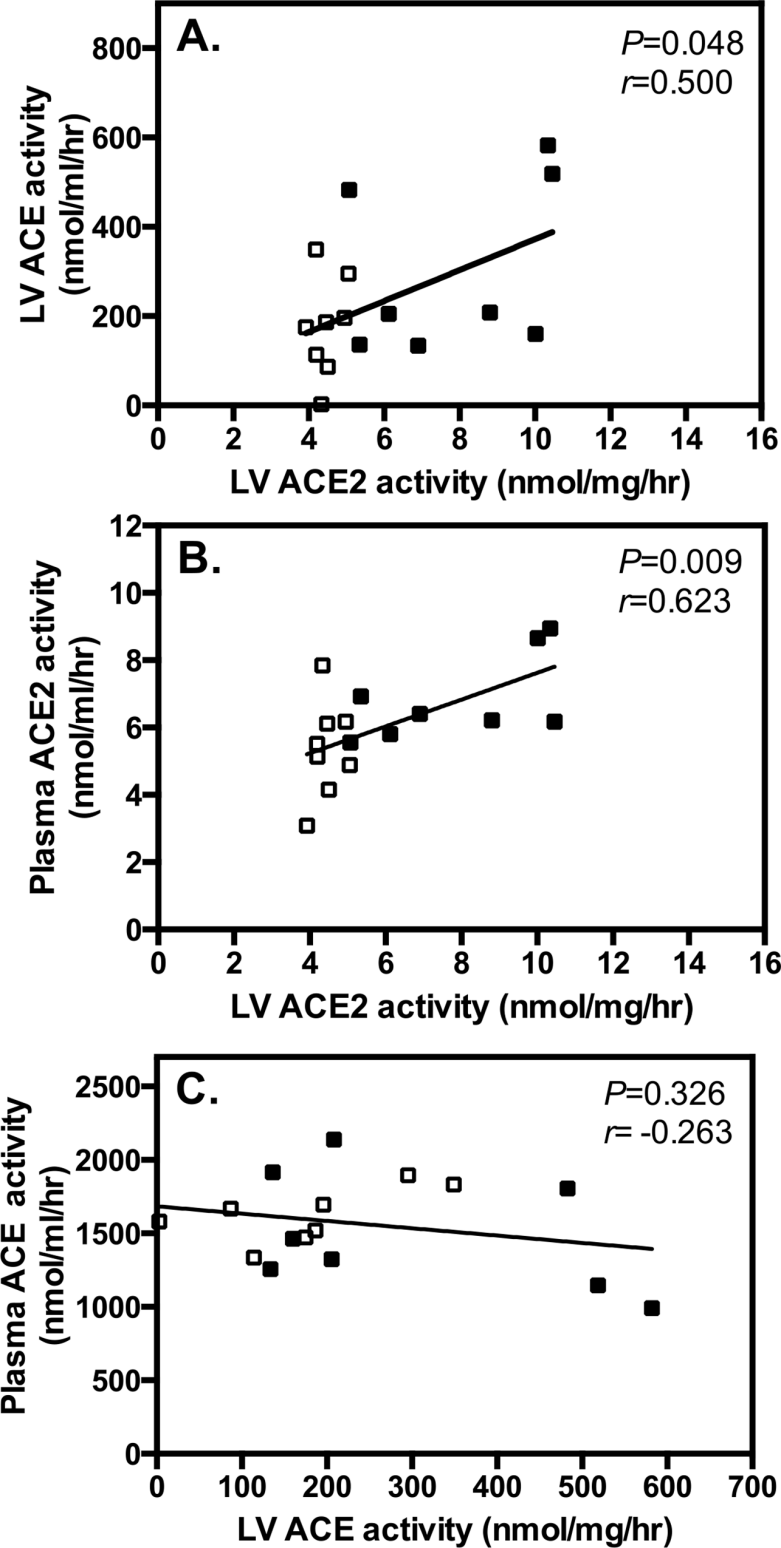
Cardiac ACE2 activity is increased to counteract elevated cardiac ACE and is shed into the circulation. Correlation analysis shows increased left ventricular (LV) ACE2 activity is associated with increased LV ACE activity (**A**) and plasma ACE2 activity (**B**), with no correlation between LV tissue and plasma ACE. Control and subtotal nephrectomy (STNx) rats without active treatment were used for the correlation analysis. Open squares represent Control rats; closed squares represent STNx rats.

In vehicle-treated STNx rats increased cardiac ACE2 activity correlated with increased cardiac ACE activity (Fig 4A), which supports the notion that in disease ACE2 increases to balance the effects of elevated cardiac ACE. The correlation between increased cardiac ACE2 and increased plasma ACE2 activity (Fig 4B) taken together with upregulation of cardiac ADAM17 mRNA suggests that increased ACE2 shedding from the diseased heart may be responsible for the increase in circulating levels of ACE2. By contrast, there was no correlation between LV ACE and plasma ACE activity.

#### *In vitro* effect of DIZE on cardiac ACE2 activity, and ACE activity and binding

**Fig 5:**
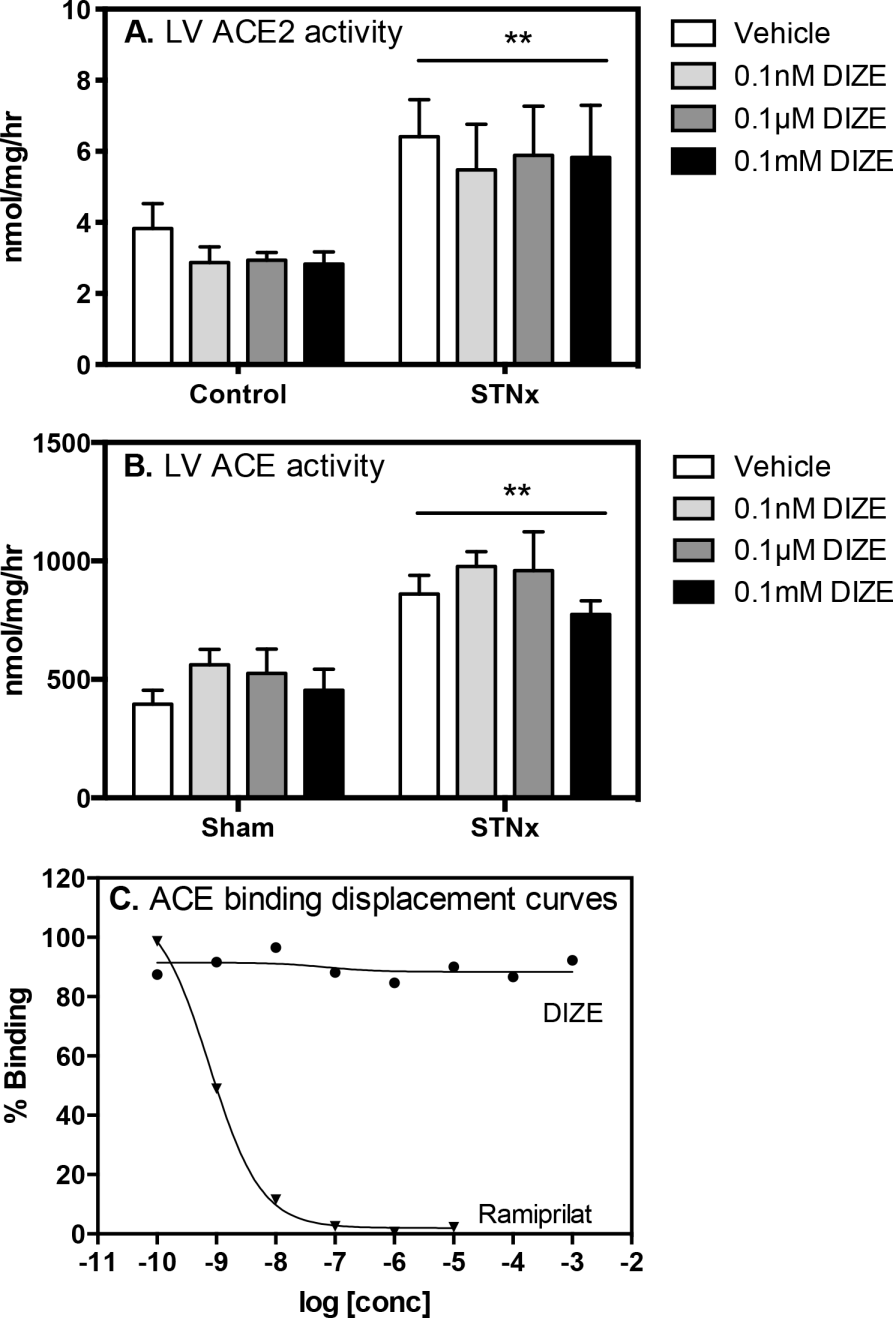
DIZE does no effect cardiac ACE and ACE2 under *ex vivo* conditions. *Ex vivo* DIZE had no effect on endogenous ACE2 (**A**) and ACE (**B**) activity in LV membrane preps (100μg per well) from Control (*n* = 4) and subtotal nephrectomy (STNx, *n* = 4) rats. The ACE inhibitor, ramiprilat caused a concentration-dependent displacement of specific ^125^I-MK351A binding from rat LV ACE and DIZE had no effect on ACE binding (**C**).

The *ex vivo* effect of DIZE (0.1mM, 0.1μM, 0.1nM) on LV ACE2 and ACE activity was measured in Control and STNx rats. As shown in Fig 5, DIZE had no effect on cardiac ACE2 (Fig 5A) or ACE activity (Fig 5B) in either Control or STNx rats. The *ex vivo* effect of DIZE and ACE inhibition with ramiprilat on cardiac ACE was also examined; ramiprilat inhibited cardiac ACE but DIZE had no effect on ACE binding (Fig 5C).

## Discussion

ACE2 is highly expressed in the heart and is an important regulator of cardiac function [31, 32]. We explored the role of ACE2 in the cardiac consequences of kidney disease using a model of kidney injury due to STNx. The results confirm our previous reports that STNx leads to LVH, impaired cardiac function and elevations in cardiac ACE and ACE2 activity [19, 20]. We also report that STNx rats had increased cardiac Ang II and Ang 1–7 levels and increased cardiac gene expression of ADAM17.

The results of this study were that a 2 week s.c. infusion of DIZE significantly improved diastolic function and cardiac fibrosis in STNx rats, and these benefits were achieved in the absence of a fall in blood pressure or any improvement in kidney function. DIZE reduced the gene expression of BNP, an indirect marker of cardiac injury in STNx rats, and shifted the cardiac RAS balance to a more cardioprotective profile with a reduction in both cardiac ACE and cardiac Ang II. There was no change in cardiac ACE2 activity or in cardiac Ang 1–7 levels with DIZE. The prevailing Ang II levels represent a balance between formation (due to ACE), and degradation (due to ACE2). We found no evidence that DIZE increased cardiac Ang 1–7 levels, suggesting that the major benefit of DIZE is due to reduced Ang II formation.

It is unclear how DIZE is mediating its effects in STNx rats. The *in vitro* studies showed no direct effect of DIZE on ACE2 or ACE activity. *In vivo*, the effect of DIZE on cardiac ACE2 contrasts with our previous results where the cardiac benefits of ACE inhibition were associated with a *reduction* in cardiac ACE and ACE2 [13]. The finding that DIZE was associated with down regulation of ADAM17 mRNA led us to speculate that DIZE may have indirect effects to maintain cardiac ACE2 activity levels and thus reduce cardiac Ang II, through reduced cleavage of ACE2 from cardiac cells. This hypothesis would be consistent with reports that cardiomyocyte-specific deletion of ADAM17 prevented shedding of ACE2, whilst exogenous infusion of Ang II increased myocardial ADAM17 expression and decreased myocardial ACE2 activity [33]. Future studies should address not only ADAM17 gene expression but also ADAM17 protein content.

Our results also show that the relative balance or imbalance of the ACE/Ang II and the ACE2/Ang 1–7 pathway may determine the *in vivo* effect of DIZE on the underlying pathophysiology. Thus, by contrast to its effects in the pathological state of kidney failure, DIZE had no effect on cardiac ACE/ACE2 activity, cardiac Ang peptides or ADAM17 in Control rats with a balanced RAS and normal blood pressure and kidney function. We have previously shown that short-term DIZE significantly reduced kidney cortical ACE activity and ameliorated the reduction in cortical and medullary ACE2 activity in STNx rats with kidney RAS imbalance, but had no effect on kidney ACE2 expression or activity in Control rats with a balanced RAS [13]. Further support for this concept comes from studies of DIZE after myocardial infarction (MI) which is also associated with imbalance of the cardiac RAS [22]. A 4-week s.c infusion of DIZE in MI in rats improved cardiac remodelling, significantly increased cardiac ACE2 mRNA and activity and reduced cardiac ACE mRNA and activity–these effects were blocked by concurrent use of the ACE2 inhibitor, C16 [22]. In hypercholesterolemic mice on a wild-type or ACE2 deficient background with Ang II induced abdominal aortic aneurysms [34], 4-weeks of intramuscular DIZE (30mg/kg) increased kidney ACE2 mRNA and activity in wild-type mice, and reduced the incidence and severity of Ang II-induced abdominal aortic aneurysms. However DIZE had no effect in ACE2-deficient mice which does suggest that it acts through an ACE2-dependent mechanism [34].

The precise mechanism of action of DIZE remains unclear [35] and there is conflicting evidence with regard its effect on ACE2 activity [13, 15, 23, 35, 36]. Kulemina *et al* first reported the off target effects of DIZE to activate ACE2 [15] and described a biphasic dose–response curve; DIZE activated ACE2 at low concentrations and partially inhibited ACE2 at high concentrations. Incubation of human rACE2 with DIZE (100μM) increased ACE2 activity in some studies [23], but others have shown that DIZE did not increase the enzymatic activity of mouse or human rACE2 [35]. We reported that DIZE had no effect *ex vivo* to alter kidney ACE2 activity, but was associated with increased kidney ACE2 activity *in vivo* in STNx rats [13]. The *ex vivo* studies in cardiac membranes of STNx and control rats in the current study showed no direct effect of DIZE to increase ACE2 activity. Taken together, the reported effects of DIZE on ACE2 activity vary according to whether the study was *in vivo* or *ex vivo*, and may depend on the underlying pathophysiology, route of administration or the tissue used to assess ACE2 activity. In order to delineate if the mechanism of action of DIZE is specifically related to ACE2 activation, future studies should examine the effect of co-administration of DIZE with specific ACE2 inhibitors such as MLN 4760 (C16) to help establish or exclude if the observed beneficial cardiac effects of DIZE are truly ACE2 dependent.

With regard to ACE, the reduction in cardiac ACE activity in STNx with DIZE is likely to be an indirect effect due to reduced cardiac damage and thus less ACE activation. The degree of ACE “inhibition” with DIZE is less than that would be expected with an ACE inhibitor [14, 19], and ACE inhibition usually results in a fall in blood pressure. The *ex vivo* studies in cardiac membranes showed no direct effect of DIZE to inhibit ACE, which is consistent with the work of others that DIZE had no effect on the catalytic activity of ACE [15].

In summary, short-term intervention with the presumed ACE2 activator DIZE ameliorates cardiac dysfunction and cardiac fibrosis in STNx rats. The benefits of DIZE were associated with a shift in the cardiac RAS balance to a cardioprotective profile with reduced ACE and Ang II and no change in cardiac ACE2 activity. Cardiac Ang 1–7 levels were not changed with DIZE suggesting that its main effects are related to reduced Ang II. We have previously reported in STNx, that improved cardiac structure and function was associated with reduced cardiac ACE2 activity, and the finding that cardiac ACE2 remained unchanged with DIZE may reflect reduced cardiac ACE2 shedding secondary to downregulation of cardiac ADAM17. Further studies are now warranted to determine if the observed beneficial cardiac effects of DIZE are due to reduced ACE2 shedding from the cell membrane, or are specifically related to ACE2 activation.

Studies are also needed to assess the long-term impact of DIZE on both cardiac and kidney function, and to investigate if combining DIZE with standard RAS blockade has incremental effects to improve the cardiac consequences of kidney disease or to prevent progression to chronic kidney disease. Although DIZE is used for the treatment of trypanosomiasis or sleeping sickness, side effects may preclude its widespread clinical use [37–39] and specific compounds that selectively amplify ACE2 activity will be needed if this approach is to find utility.

## Supporting Information

S1 Fig ACE2 protein expression increases in STNx. Left ventricular (LV) ACE2 protein expression in Control (Cont) and subtotal nephrectomy (STNx) rats (*n* = 8/group). Right hand panel consists of representative photomicrographs of ACE2 immunohistochemical labelling (brown staining) (magnification x200). Data expressed as mean ± SEM. *P<0.05 disease effect (Control vs. STNx)
(TIFF)
