# Supplementary figures and images for: Diminazene Aceturate Improves Cardiac Fibrosis and Diastolic Dysfunction in Rats with Kidney Disease
Source: PLoS One. 2016 Aug 29;11(8):e0161760. doi: 10.1371/journal.pone.0161760 (PMC5003360; doi:10.1371/journal.pone.0161760)

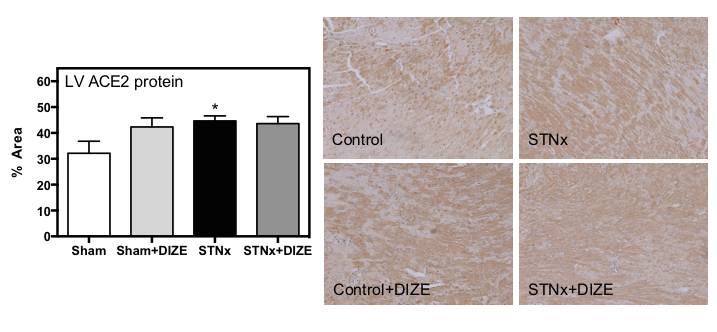

Supplement: S1 Fig — Left ventricular (LV) ACE2 protein expression in Control (Cont) and subtotal nephrectomy (STNx) rats (n = 8/group). Right hand panel consists of representative photomicrographs of ACE2 immunohistochemical labelling (brown staining) (magnification x200). Data expressed as mean ± SEM. *P<0.05 disease effect (Control vs. STNx) (TIFF) [file pone.0161760.s001.tiff]
